# Coevolution of Human Diet and Gut Microbiome: Implications for Nutrigenomics and Cross‐Population Health

**DOI:** 10.1155/ijm/5597426

**Published:** 2026-04-27

**Authors:** Ferry Sandra, Alifah Evi Scania, Nurrani Mustika Dewi, Dewi Ranggaini, Johni Halim, Alfred Pakpahan, Kyung Hoon Lee

**Affiliations:** ^1^ Department of Biochemistry and Molecular Biology, Division of Oral Biology, Faculty of Dentistry, Universitas Trisakti, Jl. Kyai Tapa No. 260, Jakarta, 11440, Indonesia; ^2^ Faculty of Dentistry, Center of Molecular Biology Study, Universitas Trisakti, Jl. Kyai Tapa No. 260, Jakarta, 11440, Indonesia; ^3^ The Prodia Education and Research Institute, Jl. Kramat Raya No. 150, Jakarta, 10430, Indonesia; ^4^ Department of Physiology, Division of Oral Biology, Faculty of Dentistry, Universitas Trisakti, Jl. Kyai Tapa No. 260, Jakarta, 11440, Indonesia; ^5^ Department of Biology, Division of Oral Biology, Faculty of Dentistry, Universitas Trisakti, Jl. Kyai Tapa No. 260, Jakarta, 11440, Indonesia; ^6^ Research Institute, Ballys Co., Ltd., Incheon, 22219, South Korea

**Keywords:** coevolution, cross-population health, gut microbiome, nutrigenomics, personalized nutrition

## Abstract

The coevolution of the human diet and gut microbiome has played a pivotal role in shaping metabolic, immune, and epigenetic functions across human history. Dietary transitions from high‐fiber ancestral patterns to modern ultraprocessed diets have markedly influenced microbial diversity and functionality, contributing to the emergence of chronic diseases such as obesity, Type 2 diabetes, and inflammatory conditions. Recognizing the significance of gut microbial patterns in humans, this review explores the coevolution of diet and gut microbiota, especially on how gut microbiota influences human gene regulation, and the implications of these interactions for personalized nutrition and global health strategies. Comparative insights across populations in different periods reveal that geography, dietary practices, and host genetics interact to shape distinct microbiome configurations and disease susceptibility. Therefore, implementing a nutrigenomics and nutrigenetics approach might provide a molecular framework to understand these interactions and to develop personalized nutrition strategies. Though several clinical implementations utilizing genomic data have been embedded in several countries, global implementation remains challenging due to population‐specific genetic variability, cultural dietary preferences, cost limitations, and ethical considerations. Integrating microbiome and genetic data into clinical practice and public health policy offers a promising path to mitigate diet‐related health disparities that is tailored to individual and population‐level needs.

## 1. Introduction

The human microbiota are structured by their biological interactions with the host [1], hence forming an ecosystem that has evolved over millennia. While it has been well known that genetic background, geographical localization, and culture might influence microbiota composition in the human body, diet plays a major role in restructuring the microbial composition [2]. Diet is the most potent environmental factor shaping gut microbial composition, accounting for 5–20% of interindividual variation [3]. Coevolution of human diet and the gut microbiome represents a profound symbiotic relationship that has shaped physiology and health outcomes across human history [4].

During the era of the agricultural revolution, also known as the Neolithic transition, human diets gradually shifted in many regions, including Europe and North Africa. The diet during this era was mostly obtained from wild, often marine or freshwater resources, but the adoption of farming and animal domestication later pushed the intensification of crop–livestock systems, shifting the dietary pattern to terrestrial crops and livestock [5–8]. During urbanization, following the rural‐to‐urban migration, changes in the food environment, rising incomes, and time constraints have caused dietary patterns to shift from staple‐based foods to more animal‐source, sugary, processed, and convenience foods [9–12]. This alternation caused several microbiome disruptions and raised the risk of overweight, obesity, and cardiometabolic disease [11, 13, 14]. During the Industrial Revolution, especially in Western Europe, the diets were low in total calories and dominated by starchy staple foods, such as bread and potatoes [15]. During this era, due to the industrial growth, rising real incomes, and new trade in sugar and oils, the dietary patterns later shifted to diversified diets richer in animal products, fats, and sugar [15–17].

However, the most dramatic changes have occurred in the post–World War II era, which was marked by the global industrial food systems, ultraprocessed foods (UPFs), rising animal products and sugar, emerging alternative proteins, high refrigeration utilization, and dietary fiber intake decline [18–20]. The Western dietary pattern was known to include high sugar, fat, and additives, as well as low microbial‐accessible carbohydrates that became dominant in highly industrialized nations [21, 22]. While these transitions reduced some micronutrient deficiencies and enabled higher average energy intake, they contributed to a surge in obesity and diet‐related noncommunicable diseases [10, 19]. This dietary pattern caused a dominance of *Bacteroides* that is associated with inflammation and metabolic dysfunction [23]. This lower fiber intake also leads to a decline in microbial diversity [13, 14], which correlates with a rise in chronic inflammatory conditions such as metabolic syndrome, inflammatory bowel disease (IBD), oral, and colorectal cancers [24, 25]. In addition, additives introduced in the mid‐20th century, such as emulsifiers, also disrupt gut barriers, worsen inflammation, and increase disease susceptibility [26].

The human gut microbiome, which functions as a metabolic engine and produces short‐chain fatty acids (SCFAs) that supply energy, reduce inflammation, and help regulate blood sugar [27, 28], is influenced by diet. Microbes can impact gene expression by modifying deoxyribonucleic acid (DNA) markers related to metabolism and immunity as well, highlighting the direct effect of diet on our biology through microbial activity [28, 29]. Recent metagenomic studies reveal the gut microbiome as a dynamic metabolic organ encoding over 3 million genes, far exceeding the human genome’s 20,000 genes [30]. The microbiota metabolizes indigestible polysaccharides into SCFAs, synthesizes essential vitamins, and modulates host gene expression via epigenetic mechanisms [31, 32].

However, genetic variations, in turn, affect how certain foods impact the microbiota and contribute to health outcomes [33]. Building on these findings, the rise of nutrigenomics, an interdisciplinary field exploring how food components interact with gut microbes and host genes, has gained momentum [34]. However, interindividual responses to these dietary components vary widely, influenced by differences in genetic background, diet, and baseline gut microbiome composition, which together explain a significant portion of the variability in host metabolic responses [35]; therefore, it is interesting to be explored further. This review explores the coevolution of diet and gut microbiota, highlighting how dietary changes reshape microbial communities, how microbial products influence human gene regulation, and the implications of these interactions for personalized nutrition, global health, and strategies to combat diet‐related diseases.

## 2. Coevolution of Human Diet and the Gut Microbiome

Human diets and gut microbiomes have coevolved across three distinct epochs: preindustrial (before 1750), industrialized (1750–1945), and post–World War II (post‐1945) (Table 1). In ancient hunter‐gatherer, such as the Hadza population, their diets are rich in wild plants and fiber; hence, their gut microbiomes are dominated by Firmicutes (72 ± 1.9%) and Bacteroidetes (17 ± 1.1%) phyla. Among the Bacteroidetes, the fiber‐degrading taxa like *Prevotella* and *Treponema*, which are key players in breaking down complex carbohydrates, were found to be dominant in abundance. Meanwhile, during the same era, in other populations, *Fibrobacter* was found to be dominant [36].

**TABLE 1:** Gut microbiome shifts across dietary epochs.

Parameter	Preindustrial (pre‐1750)	Industrialized (1750–1945)	Post–World War II (1945–present)
Fiber intake	Very high (> 100 g/day) [34]	Marked decrease (substantial reduction) [34]	Low (< 20–30 g/day in Western societies) [34]
Dominant genus	Fiber‐degrading *Prevotella*, *Treponema* [35]	Mixed profiles with less fiber degraders [35]	*Bacteroides*‐dominated [35]
SCFA production	High (due to abundant fiber fermentation) [36]	Moderate‐declining (less fiber ⟶ fewer SCFAs) [35]	Low‐moderate (Western diets ⟶ reduced SCFAs) [35]
Obesity prevalence	Minimal or rare (< 1%) across populations [37]	Rising modestly as industrialization progressed, especially in wealthy nations [36]	High in high‐income countries (30%–40%) [36]
Microbial gene richness	Very high (ancestral microbiome diversity) [35]	Moderate (loss begins with industrialization) [3]	Significantly reduced (modern dysbiosis) [3]

Abbreviation: SCFA, short‐chain fatty acids.

However, industrialization in 1900 initiated a gradual decline in dietary diversity, with the increasing use of refined grains and sugar contributing to a reduction in average fiber intake [37]. Hence, the dominant gut profile is *Bacteroides*‐centered, with *Bifidobacterium*, *Ruminococcus*, *Faecalibacterium*, *Alistipes*, *Bilophila*, and *Blautia* commonly enriched, especially in low‐fiber Western populations. Meanwhile, the abundance of fiber‐degrading microbes such as *Prevotella* and diverse fiber‐fermenting *Bacteroidales* is reduced [38, 39]. For the European population, the processed sugar intake also increased significantly, contributing to a decline in *Fibrobacter* [40].

The post–World War II era marked a global inflection point in food systems, as UPFs became dietary staples. Between 1945 and 1970, refrigeration and industrial agriculture enabled the mass production of shelf‐stable, calorie‐dense foods. By the 1960s, average fiber intake in Western nations declined to approximately 11–15 g/day, while saturated fat consumption increased, contributing to a higher proportion of daily caloric intake [41]. These changes reshaped gut microbiomes within decades, not only in Western countries but also in Asia. Though direct microbiome surveys from Asia before 2000 are limited, the available studies show that Westernization has also driven Asian gut communities from diverse, fiber‐adapted, *Prevotella*‐rich configurations toward lower diversity, *Bacteroides*‐dominated microbiomes with reduced fermentative capacity and higher metabolic disease risk [42–44]. Meanwhile, inflammation‐associated *Bacteroides* species became more prevalent in Western microbiomes during this era [36]. Increased consumption of processed foods and reduced fiber intake have also been linked to an increase in IBD incidence in high‐income countries. The result of a meta‐analysis on 9 studies from different countries according to PRISMA guidelines showed that the Western dietary pattern was associated with a relative risk of 1.92 (95% CI 1.37–2.68) for all IBD, suggesting nearly a twofold increase in risk [41].

By 2000, UPFs had made up over 50% of daily energy intake in industrialized countries like the U.S. and the U.K. High UPF consumption has been linked to lower gut microbiome diversity and a reduction in *Faecalibacterium prausnitzii*, a key bacterium for gut health [45]. Urbanization further accelerated these trends: Rural Chinese populations retained 30% higher microbial diversity than urban counterparts in 2020, but rapid urbanization post‐1990 halved this gap [14, 46]. Similarly, Japan’s post‐1980 dietary Westernization showed increased Bacteroides in urban microbiomes, paralleling a rise in metabolic syndrome such as diabetes mellitus [47, 48].

Western dietary patterns disrupt this symbiosis, driving dysbiosis‐linked pathologies [24]. Individuals with *Bacteroides*‐dominant gut microbiomes, often linked to diets high in animal protein and low in fiber, may have a higher risk of metabolic syndrome compared to those with *Prevotella*‐rich microbiomes [1, 21]. Notably, polyphenol‐rich diets, such as those high in green tea, counteract these effects by reducing *Firmicutes/Bacteroidetes* and lowering systemic inflammation [49, 50]. These insights emphasize the potential of dietary interventions to recalibrate microbial–immune–metabolic crosstalk.

## 3. Molecular Interactions Between Diet, Microbiome, and Host Physiology

**FIGURE 1:**
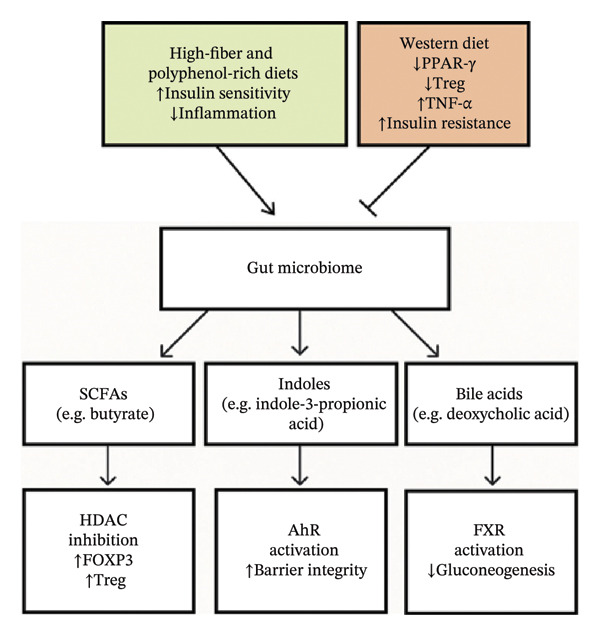
Interplay between diet, gut microbiota, and host physiology. High‐fiber and polyphenol‐rich diets promote beneficial gut microbial functions that enhance insulin sensitivity and reduce inflammation. In contrast, Western diets are associated with reduced PPAR‐γ expression, decreased regulatory T cells, elevated TNF‐α levels, and increased insulin resistance. The gut microbiome produces SCFAs, indoles, and bile acids, which modulate host metabolism and immunity through HDAC inhibition, AhR activation, and FXR activation.

The gut microbiota functions as a metabolic interface between diet and host physiology by fermenting dietary fibers into SCFAs, primarily acetate, propionate, and butyrate (Figure 1). These SCFAs influence host metabolism and immune function largely through activation of G protein–coupled receptors (GPRs), particularly GPR43 and GPR41, to regulate systemic processes including glucose homeostasis and inflammation [51]. Among SCFAs, butyrate has been shown to enhance insulin sensitivity and support glucose uptake via GPR41 activation [52]. In contrast, diets low in dietary fiber, such as typical Western diets, are associated with reduced SCFA production, which compromises anti‐inflammatory signaling and immune regulation [53]. Lower SCFA availability has been linked to impaired regulatory T cell (Treg) responses and diminished anti‐inflammatory cytokine activity, promoting intestinal and systemic inflammation [54]. These findings underscore the critical role of dietary fiber in maintaining immunometabolic equilibrium.

Beyond metabolic regulation, SCFAs, especially butyrate, also influence host gene expression through epigenetic mechanisms. Butyrate were shown to be able to modulate chromatin structure by inhibiting histone deacetylases (HDACs), thereby promoting genes involved in immune tolerance, including those supporting Treg differentiation [27, 55]. Conversely, low‐fiber diets may alter epigenetic regulation of key metabolic genes, contributing to dysregulated adipose tissue function [56]. These mechanisms illustrate how diet‐driven shifts in microbial metabolism can reshape host physiology at the molecular level (Figure 1).

Microbial metabolism of dietary tryptophan generates bioactive compounds such as indoles, which play an important role in linking diet to host physiology. Indole‐derived metabolites, including indole‐3‐propionic acid (IPA), interact with host signaling pathways to support intestinal barrier function, in part through the activation of the aryl hydrocarbon receptor (AhR). Through this signaling axis, indoles contribute to the maintenance of epithelial integrity and protection against increased intestinal permeability [57, 58]. In parallel, gut microbes also modify primary bile acids into secondary bile acids, which act as signaling molecules influencing host metabolic regulation. Secondary bile acids such as deoxycholic acid, synthesized by *Clostridium scindens*, regulate glucose metabolism via activation of the farnesoid X receptor (FXR), thereby modulating glucose metabolism and hepatic energy balance [59, 60]. Alterations in bile acid profiles have been associated with metabolic disturbances, particularly in the context of obesity [60]. Together, these pathways underscore the microbiome’s role as a biochemical translator of dietary signals.

## 4. Population Comparisons: Asian, Western, and African Populations

The gut microbiome’s composition and functional potential vary significantly across populations, shaped by geography, dietary practices, and host genetics. In Asian populations, especially in South Korean, they used to consume traditional Korean diets that are characterized by plant‐based and fermented foods that are low in fat and rich in carbohydrates, plant protein, vitamins, minerals, and fiber [61]. In a study, in Korean adult population, the gut microbiome is predominantly composed of Bacteroidetes (48.8%) and Firmicutes (42.8%), with dominant genera *Bacteroides*, *Prevotella, Faecalibacterium*, *Oscillospira*, and *Ruminococcus* [62]. In the Japanese population that also often consumed fermented foods like natto, in a cohort study, it was found that the gut’s microbiomes were mostly Firmicutes and Bacteroidetes‐dominant, with predominant genus *Bacteroides*, *Bifidobacterium*, *Faecalibacterium*, *Blautia*, *Ruminococcus*, *Roseburia*, and *Prevotella* [63], which is quite similar to the South Korean population. In a cohort study examining the Chinese population, it was also found that Firmicutes and Bacteroidetes are the predominant phyla in the Chinese’s gut microbiome. *Prevotella* and *Bacteroides* are recognized to be highly dominant along with several other genera such as *Escherichia*, *Bifidobacterium*, *Blautia*, *Faecalibacterium, Ruminococcus, Alistipes,* and *Roseburia* [64]. From these studies, it can be seen that most East Asian countries that still consume traditional foods mostly share a similar microbial profile. *Prevotella* and *Bacteroides,* which are found in all three populations, enhance fiber fermentation, resulting in elevated SCFA levels, such as butyrate [65]. Meanwhile, *Bifidobacterium*, *Faecalibacterium*, and *Roseburia* are also known to be SFCA producers. This microbial profile is often correlated with low obesity rates (< 5% BMI ≥ 30 kg/m^2^) and is associated with metabolic health (Table 2) [69, 70].

**TABLE 2:** Classified obesity rates and SCFA levels by population.

Population	SCFA level	Obesity rate (% BMI ≥ 30)	Classification
Rural Asian	Not directly quantified; presumed moderate–high due to high‐fiber intake	5%–10% [66]	Moderate–high SCFA, normal obesity [66]
Urban Asian	↓ SCFA, esp. butyrate, in obese individuals [67]	16% [67]	Moderate SCFA, overweight [67]
Western	10–25 mmol/kg fecal SCFAs [68]	30%–40% [68]	Moderate SCFA, obese [68]
Rural African	Highest SCFA in metabolic syndrome study cohorts [68]	6% [68]	High SCFA, normal obesity [68]
Urban African	↓ SCFA (esp. acetate and butyrate); inverse with BMI [68]	40% [68]	Low SCFA, obese [68]

Abbreviations: BMI, body mass index; SCFA, short‐chain fatty acids.

In a multi‐Asian country study, it was reported that Malaysian, Indian, and Chinese gut microbiomes are dominated by Bacteroidetes, Firmicutes, and Actinobacteria, while Thai populations are more Firmicutes‐dominated with a smaller abundance of Bacteroidetes [71]. Thailand’s gut microbiomes are quite different from those in East Asian countries, since they are mostly dominated by Clostridiales (> 25%) and Lachnospiraceae, but *Ruminococcaceae* is also found to be quite abundant, just like other Asian countries [71, 72]. As for eastern and southeastern Asian countries, global countries found that Firmicutes is the most prevalent phylum across all regions, and the samples from Central and Southern Asia exhibit higher relative abundances of Actinomycetota and lower abundances of *Bacteroides* compared to other regions, with high *Prevotella* [73]

However, urbanizing Asian populations adopting Western diets are now experiencing a 15–20% annual rise in Type 2 diabetes mellitus incidence [66]. This increase has been linked to a 40% reduction in *Akkermansia muciniphila* abundance and impaired gut barrier function [67]. Targeted interventions such as fiber supplementation (30 g/day) in high‐risk Asian populations have restored microbial diversity by 25% and lowered HbA1c levels, highlighting the promise of precision nutrition [68, 74]. This Westernization is also shown by the reduced proportion of Prevotella in some Asian countries, such as in South Korea [61].

In Western populations, diets are typically high in fats and added sugars, and are associated with gut microbiomes dominated by *Bacteroides*, which show reduced microbial gene richness. This microbial configuration was associated with a higher prevalence of metabolic syndrome [21]. Beyond diet, host genetics also shape microbial ecology. Lactase persistence, a polymorphism prevalent in ≥ 60% of northern Europeans, facilitates dairy consumption and supports the growth of *Bifidobacterium* that metabolize lactose into anti‐inflammatory SCFAs [32]. Additionally, variants in *APOA2* (rs5082), common in Mediterranean populations, can exacerbate obesity risk under high‐saturated fat diets via altered lipid absorption and an increase in *Clostridium* abundance [75]. Clinically, African‐Americans in the US exhibit a higher incidence of IBD compared to Caucasians, a disparity linked to the overgrowth of *Bacteroides vulgatus* and diminished SCFA production [76].

By contrast, African populations such as the Hadza of Tanzania maintain ancestral fiber‐rich diets (70–120 g/day), fostering high microbial diversity dominated by *Prevotella* and *Treponema* [36]. These microbes degrade complex plant polysaccharides and produce anti‐inflammatory metabolites, supporting gut health. *Prevotella*, often associated with non‐Western microbiomes, shows higher relative abundance in sub‐Saharan Africa, Latin America and the Caribbean, and Northern Africa when compared to Europe and Northern America [73]. Rural African populations show remarkable resilience against metabolic disorders, with obesity rates below 2% [77]. However, urbanization poses a growing threat. Nigerian cities are now reporting a 10% annual increase in hypertension, which has been associated with shifts in the Firmicutes/Bacteroidetes ratio [78]. These changes underscore the fragility of traditional microbial ecosystems in the face of dietary westernization.

## 5. Nutrigenomics: Concepts and Biomolecular Applications

Through nutrigenomics, the influence of dietary components and specific food‐derived compounds on gene expression, cellular function, and physiological outcomes can be elucidated via molecular and epigenetic mechanisms [79, 80]. Nutrients such as SCFAs, polyphenols, and omega‐3 fatty acids can regulate gene activity by interacting with nuclear receptors (e.g., PPAR‐γ, AhR) or by modifying epigenetic marks such as DNA methylation and histone acetylation [81]. For example, butyrate, a microbial metabolite produced from dietary fiber fermentation, inhibits HDACs, leading to a 50% increase in acetylation at the *FOXP3* promoter and promoting the differentiation of regulatory T cells [82]. Likewise, resveratrol, a polyphenol found in grapes and berries, activates the SIRT1 deacetylase, enhancing mitochondrial function and reducing oxidative stress in high‐fat diet conditions [83, 84]. These interactions highlight the dynamic and reciprocal relationship between diet and the genome.

Nutrient‐induced changes in gene expression have been demonstrated in various physiological contexts. Consumption of resistant starch (40 g/day) has been associated with increased expression of *GLUT4* in adipose tissue by over twofold [85], alongside a reduction in the expression of the proinflammatory cytokine *TNF-α* in both obese individuals and obese mouse models, indicating an alleviation of inflammation [86, 87].

Beyond immediate gene expression, nutrients consumed in early‐life developmental process might induce long‐term regulatory effects through epigenetic modifications. For example, diets rich in folate have been shown to reduce global DNA methylation at *PPAR-γ* loci, which is associated with a lower risk of obesity [88, 89]. Similarly, in adults, nutrients might also influence epigenetic changes, though not necessarily permanently. For example, high‐fat diets promote hypermethylation at the *FTO* gene locus, contributing to an increase in leptin resistance [90]. Clinical dietary interventions, such as adherence to a Mediterranean diet for 12 weeks, have been shown to increase histone H3K27 acetylation in immune cells, with concurrent improvements in insulin sensitivity [91]. Altogether, these findings underscore the critical role of nutrition in shaping gene regulation and offer promising avenues for disease prevention and personalized nutrition strategies based on molecular responses to diet.

Incorporating epigenetic information into nutrigenetic models might hold promise, but its clinical translation remains constrained by several methodological and biological limitations. Epigenetic markers such as DNA methylation and noncoding RNAs are highly dynamic, cell‐ and tissue‐specific, and strongly influenced by age, lifestyle, medications, and gut microbiota, making it difficult to distinguish causal signals from context‐dependent noise in routine practice [92, 93] Technical variability between platforms and bioinformatic pipelines further complicates comparability across cohorts, while the need for tissue‐specific data is difficult to reconcile with reliance on peripheral blood in large human studies [92, 94]. Finally, current multiomics approaches integrating epigenetics, host genome, and gut microbiome remain complex, expensive, and not yet scalable for routine care, and the real‐world effectiveness, cost utility, and ethical implications of using epigenetic markers to guide individualized dietary prescriptions remain unexamined [34, 93, 95].

### 5.1. Nutrigenetics and Personalized Dietary Responses

While nutrigenetics focuses on how diets might induce changes in gene expression and regulation [34], nutrigenetics explores how genetic variations influence individual responses to dietary components, enabling precision nutrition strategies tailored to genetic profiles [79, 96]. Both fields are complementary and together underpin precision or personalized nutrition, but they differ in whether they start from genetic variation (nutrigenetics) or from diet‐induced changes in gene expression and regulation (nutrigenomics) [34, 97].

In nutrigenetics level, single nucleotide polymorphisms (SNPs) in genes such as methylenetetrahydrofolate reductase (*MTHFR*, C677T), *FTO* (rs9939609), and lactase (*LCT*, ‐13910C > T) significantly modulate nutrient metabolism and disease risk (Table 3). Carriers of the *MTHFR* TT genotype exhibit 20% lower serum folate levels compared to CC individuals when consuming low‐folate diets (< 255 μg/day), increasing their risk of hyperhomocysteinemia [98, 100]. Similarly, *FTO* rs9939609 AA homozygotes show greater weight gain on high‐fat diets compared to TT carriers, likely due to impaired leptin signaling [99, 101]. These genetic nuances highlight the importance of genotype‐guided dietary recommendations.

**TABLE 3:** Key nutrigenetic variants and dietary implications.

Gene	Variant	Dietary trigger	Physiological effect	Ethnic consideration
*MTHFR*	C677T	Low‐folate intake (< 400 μg/day) [87]	TT genotype associated with 20% lower serum folate and increased homocysteine, raising risk of hyperhomocysteinemia and vascular disease [87]	Prevalence varies 25%–57% in Mexican/Amerindian, 10%–35% in Europeans, < 5% in East Asians [87]
*FTO*	rs9939609	High‐saturated fat diet (≥ 30% energy from fat) [90]	A allele carriers exhibit greater adiposity, increased appetite for fat and sugar, likely via appetite/hormone pathways [90]	Risk effects observed in Europeans and Asians. MAF 14% in Chinese, 39% in Europeans [90]
*LCT*	‐13910C > T	Lactose ingestion [98]	T allele enables lactase persistence, reducing GI symptoms. Nonpersistence causes lactose intolerance [98]	High persistence in Northern Europeans (70%–90%), low (< 10%) in East Asians [98]
*APOA2*	rs5082	High‐saturated fat intake (≥ 22 g/day) [99]	CC genotype linked to greater BMI/insulin resistance under high SFA diets [99]	Observed in Europeans/Mediterranean, absent in East Asians due to lower MAF and fat intake [99]

Abbreviations: BMI, body mass index; MAF, minor allele frequency; SFA, saturated fatty acids.

Building on these genetic associations, recent advances in clinical research and technology are enabling the practical application of nutrigenetics in personalized dietary interventions [102, 103]. Individuals carrying the APOE *ε*4 allele, a genetic risk factor for hypercholesterolemia, exhibited a greater reduction in LDL cholesterol on a low‐glycemic, high‐fiber diet compared to standard dietary guidelines [104, 105]. Similarly, lactase nonpersisters receiving lactose‐free probiotics, such as *Lactobacillus acidophilus* DDS‐1, reported fewer gastrointestinal symptoms compared to placebo groups [106]. Additionally, machine learning algorithms that integrate microbiome, dietary, anthropometric, and blood parameters have been shown to accurately predict individualized glycemic responses to meals, supporting the potential of personalized nutrition strategies [107–109].

Despite the promise of machine learning prediction models in personalized nutrition, significant challenges regarding reproducibility and generalizability remain. While algorithms integrating multiomics data have demonstrated impressive predictive accuracy in controlled settings, their performance often deteriorates when applied to independent cohorts or real‐world populations. Key limitations include overfitting to training datasets, insufficient validation across diverse ethnic and demographic groups, and the lack of standardized protocols for data collection and model evaluation. Batch effects, differences in microbiome profiling methods, for example, sequencing vs. shotgun metagenomics, and variability in dietary assessment tools can substantially impact model performance and reproducibility [30, 110]. Therefore, while machine learning holds considerable potential for advancing precision nutrition, rigorous external validation, transparent reporting of model development and performance metrics, and integration of mechanistic biological insights are essential, these tools can be reliably implemented in clinical practice.

However, these genetic associations often vary across different ethnic populations due to differences in allele frequencies and gene–environment interactions. Lactase persistence (*LCT* ‐13910C > T) is present in 70%–90% of northern Europeans but less than 10% of East Asians, necessitating lactose‐free alternatives such as soy or almond milk for lactose‐intolerant populations [111]. Conversely, the Apolipoprotein A‐II (*APOA2*) rs5082 variant, which increases obesity risk on high‐saturated fat diets in Europeans, shows no effect in East Asians, likely due to differing dietary patterns [112, 113]. These disparities highlight the challenges of extrapolating Western‐derived nutrigenetic data to non‐European populations.

### 5.2. Public Health and Clinical Implications

Nutrigenomics is transforming preventive care by enabling the early identification of genetic susceptibilities to diet‐related diseases. Carriers of the APOE *ε*4 allele found in 15%–25% of the global population experience a reduction in LDL cholesterol when prescribed a Mediterranean diet tailored to their genotype, thereby lowering cardiovascular risk [104]. Similarly, polygenic risk scores (PRS) that incorporate obesity‐linked SNPs can identify individuals at increased risk of weight gain on Western diets, supporting more targeted dietary counseling [114]. PRS‐guided interventions have also been shown to reduce the incidence of Type 2 diabetes in high‐risk groups, with corresponding decreases in HbA_1c_ levels [115]. Collectively, these findings underscore the potential of nutrigenomics to shift healthcare from reactive treatment to proactive wellness strategies.

Building on these clinical advances, nutrigenomics is also shaping public health policy through the integration of omics data into national dietary frameworks [116]. Current Japanese dietary recommendations encourage the daily consumption of fermented foods such as natto and miso to increase *Bifidobacterium* abundance, a factor associated with a lower incidence of colorectal cancer [117]. Since *Bacteroides*‐dominant microbiomes have been considered as a risk factor for metabolic syndrome, the United States National Academy of Medicine recommends a fiber intake of 30–38 and 21–25 g per day for men and women, respectively [118]. Despite these advancements, few national dietary guidelines currently incorporate genetic or microbiome data, highlighting a significant gap between emerging nutritional science and public health recommendations.

Building on these policy‐level shifts, recent clinical trials conducted across Asia further highlight the growing global relevance of nutrigenomics in diverse populations. In China, a randomized controlled trial demonstrated that a personalized nutrition intervention tailored to individual genetic, phenotypic, and lifestyle profiles [119]. This might lead to improved anthropometric and metabolic outcomes in overweight adults, including reductions in BMI, waist circumference, and blood lipid levels [119–122]. In South Korea, bio‐germanium supplementation significantly enhanced immune function by increasing natural killer (NK) cell activity and IgG1 levels, suggesting a nutrigenomic mechanism of immunomodulation linked to mineral intake [123]. India’s NUDGE trial (CTRI/2021/09/036121) is currently evaluating gene‐based dietary advice for individuals with Type 2 diabetes, using *TCF7L2* and *PPARG* variants to guide nutritional recommendations aimed at improving glycemic control [124]. Meanwhile, in Indonesia, studies have explored gene–nutrient interactions in early childhood. One trial investigated how dietary protein and calcium influence *mTOR* gene expression and linear growth in school‐aged children (NCT03895151) [125], while another assessed how iron and DHA intake modulate gene expression and cognitive development in toddlers (NCT01504633) [126]. Although some of these studies are ongoing or have unpublished results, collectively they emphasize the need to localize nutrigenomic research within specific genetic and nutritional contexts. These findings demonstrate that precision nutrition is not only a tool for individualized healthcare but also a scalable strategy to address region‐specific health disparities in underrepresented populations.

Meanwhile, in Italy, the applications of genomics, epigenetics, nutrigenomics, and microbiome have been integrated into national regulations and governance tools. They used the national‐level incorporation of omics into prevention and health planning, for example, by implementing early modification of microbiota in disease before its clinical onset [127]. A meta‐analysis reviewing Western diet’s global impacts on metabolism and health based on studies from various countries recommends several public health policy recommendations can be offered, focusing on promoting healthier dietary habits and increasing physical activity. Recommendations for policymakers that can also be implemented are regarding the food labeling and taxation to inform consumers on the nutritional content of each food and to warn them about unhealthy, processed, and refined foods [128]. While some national policies regarding the use of nutrigenomics to improve health have been applied in some countries, the embedding process of using nutrigenomics as evidence‐based tools for international public health policy is still in an early, experimental phase, constrained by cost, methods, health disparities, and governance challenges [129, 130].

Significant challenges also remain in translating nutrigenomic research into broad public health applications, particularly due to genetic diversity and socioeconomic disparities (Table 4). Allele frequencies for nutrigenetic variants, such as *FTO* rs9939609, vary considerably across populations, and the A allele is present in 45% of Europeans but only 12% of East Asians, complicating the development of universal dietary guidelines [99]. Beyond genetic variability, disparities in technological access, especially in low‐income countries, further hinder implementation. Although sequencing costs have dropped dramatically to $0.01 per megabase as of 2023, only a few from low‐income countries possess the infrastructure required for large‐scale omics profiling, limiting their ability to leverage genomic data for public health [131]. Ethical and cultural barriers also present significant challenges. Privacy concerns remain prominent, with many individuals in Asia opposing sharing genomic data [132]. Cultural dietary preferences can also impact intervention uptake; lactose‐free probiotics like *Lactobacillus rhamnosus* GG face low adoption in dairy‐centric regions such as Scandinavia, where dairy consumption is deeply embedded [133]. Navigating these multifaceted barriers is essential for the successful global implementation of nutrigenomics.

**TABLE 4:** Key challenges and solutions in nutrigenomic implementation.

Challenge	Data/example	Proposed solutions
Genetic diversity	*APOA2* rs5082 increases obesity risk in Europeans but not Asians [99]	Develop region‐specific PRS models
Ethical concerns	Many Asians oppose genomic data sharing [116]	Strengthen data anonymization protocols

Abbreviation: PRS, polygenic risk score.

Based on this review, it can be suggested that data generated through several genomic initiatives, such as the Human Microbiome Project by the USA National Institutes of Health, might support in collecting evidence regarding the positions of the gut microbiome as a critical interface between diet, host genetics, and chronic disease risk [134], thereby supporting its integration into precision nutrition and clinical care. Since microbial composition and function are highly responsive to dietary exposures, particularly fiber diversity and overall dietary patterns, therefore reinforcing patient counseling program, the clinical primacy is suggested by utilizing these genomic data. This diet and nutrigenomic approach might be able to provide precise food‐based interventions by adding a layer of personalization by elucidating gene–diet interactions that shape metabolic responses, inflammatory pathways, and microbial ecology, allowing clinicians to tailor macronutrient quality, micronutrient support, and dietary tolerances according to individual genetic variability. Using this personal genomic data, microbiome‐directed therapies, including probiotics, should also be condition‐specific and grounded in evidence‐based indications rather than generalized use. Implementing computational or artificial intelligence–driven as well as *in silico* analysis–based models, such as the Korean nutrition model [135], might be suitable for population‐specific nutrition therapy, so the risk of false associations in certain populations can be reduced. Accordingly, integrating microbiome science with genomics supports a food‐first, diversity‐focused, and evidence‐based model of personalized or population‐specified nutrition that advances preventive and systems‐oriented healthcare while maintaining clinical rigor and translational feasibility.

## 6. Conclusion

The coevolution of the human diet, gut microbiota, and genome has significantly influenced our metabolic and immune health. To better understand how dietary responses vary across populations, it is essential to gather ethnicity‐specific nutrigenomic data that considers both genetic and dietary differences. Expanding clinical trials will be important to validate interventions that are tailored to an individual’s unique microbiome–genome profile. Moving forward, research should focus on identifying reliable diet–microbiome biomarkers and leveraging artificial intelligence–driven and *in silico* analyses to translate genomic and microbial insights into personalized, clinically actionable nutrition strategies.

## Author Contributions

Ferry Sandra, Alifah Evi Scania, Nurrani Mustika Dewi, and Kyung Hoon Lee conceptualized and designed the review. Alifah Evi Scania, Nurrani Mustika Dewi, Dewi Ranggaini, Johni Halim, and Alfred Pakpahan drafted the manuscript. Ferry Sandra, Dewi Ranggaini, Johni Halim, Alfred Pakpahan, and Kyung Hoon Lee contributed to literature analysis, interpretation, and critical discussion. Ferry Sandra, Alifah Evi Scania, Nurrani Mustika Dewi, Dewi Ranggaini, Johni Halim, Alfred Pakpahan, and Kyung Hoon Lee reviewed and revised the manuscript critically.

## Funding

This study received no external funding.

## Disclosure

All authors approved the final version for submission.

## Conflicts of Interest

The authors declare no conflicts of interest.

## Data Availability

Data sharing is not applicable to this article as no datasets were generated or analyzed during the current study.
